# Lipopolysaccharide binding protein (L.B.P.) – an inflammatory marker of prognosis in the acute appendicitis

**Published:** 2012-09-25

**Authors:** C Brănescu, D Şerban, C Şavlovschi, AM Dascălu, A Kraft

**Affiliations:** * IVth Surgery Clinic, University Emergency Hospital, Bucharest, Romania; ** „Carol Davila” University of Medicine and Pharmacy, Bucharest, Romania

**Keywords:** Acute appendicits, Lipopolysaccharide binding protein (LBP), anatomopathology, acute phase proteins

## Abstract

LIPOPOLYSACCHARIDE BINDING PROTEIN (LBP) is an important mediator of the inflammatory reaction. A multitude of factors can determine the genic transcription activation and the increase of the LBP in the blood and the human body humours: Il1, Il 6, lipopolysaccharides, Gram-negative bacteria, as well as non-infectious agents.

This paper is a prospective study performed on 147 patients admitted for acute appendicitis in 2010-2012 and evaluates the dynamics of LBP in acute appendicitis, by identifying the correlations between the pre- and post-operatory levels of LBP (up to 72 hours after surgery) and the anatomopathological type (i.e. catarrhal, phlegmonous and gangrenous).

The mean pre-op LBP values are significantly different as to the histopathological result (p<0,005). Among the biological inflammatory markers measured in this present study, LBP has a dynamics of its own in the catarrhal and phlegmonous appendicitis. Thus, if after the surgical removal of the infectious source, the leukocites and neutrophiles decrease 72 hrs after surgery, LBP continues an ascending curve.

The importance of this study consists in the introduction of last generation LBP-type inflammatory markers’ dosage in the cecal appendix pathology. This implementation is brand new in the Romanian surgical practice. The good correlation between the LBP pre-op values and the histopathological diagnosis of the appendicits form that we discovered during the present study opens the way to large-scale use of the biochemical dosage of LBP in the management of acute appendicitis.

**Abbreviations: **Lipopolysaccharide binding protein – LBP; Anatomopathology – AP

## Introduction

The first appendicitis performed on December 6th 1735 [**1**] by Claudius Amyand was introduced as therapeutically relevant for Amyand’s Hernia [**2**]. In time, the science has made significant progress and the inflammatory markers’ era has begun to identify increasingly detailed sequences of each stage of the surgical pathology. 

LBP is a 50-kDa polypeptide mainly synthesized in hepatocytes and is released as a 58- to 60-kDa glycoprotein into the bloodstream after glycosylation [**3**]. The LPS-binding domain has been identified in the N-terminal end of LBP comparable to the structural and functional related bactericidal permeability increasing protein (BPI) [**4**]. Both the LBP gene and the BPI gene are closely located on the chromosome 20 [**5**]. The transcriptional regulation of the LBP gene is induced by interleukin (IL)-1 alone or synergistically by IL-1 and IL-6, leading to a maximal LBP concentration within 24-48 h after stimulation, a response that can be strongly enhanced by tumor necrosis factor (TNF)-α and dexamethasone [**6**]. Other stimuli inducing LBP-synthesis in vivo include LPS, Gram-negative bacteria, and non-infectious agents such as turpentine [**7**]. The hepatic transcriptional induction of LBP is inhibited by the anti-inflammatory cytokine transforming growth factor β1 (TGF- β1) [**8**].

LBP is constitutively present in serum at concentrations of 5e15 mg/ml and rises 10- to 50-fold during the acute-phase reaction [**9**]. 

The aim of present article is to identify the role played by inflammatory markers as prognosis factors in the acute appendicitis, mainly that of LBP; the LBP monitorization and quantification in the diagnosis stage is meant to confirm the choice of the proper surgical conduct.

## Materials and method

The aim of the study was to assess, based on the available preoperatory information, the following aspects:

- the accuracy of the intra-operatory result prediction; 

- the evolution of some parameters between the admission moment and the analysis of the correlations and associations among the pre-operatory factors.

A prospective study was therefore performed, based on a sample of 147 cases of acute appendicitis operated in 2010-2012, in the 4th Surgery Clinic of the University Emergency Hospital, Bucharest. 

The pre-operatory information consisted in:

- demographic data (i.e. age, sex, place of origin);

- clinical signs (i.e. spontaneous pain or upon palpation, tenderness, muscular defense, nausea, vomiting, bowel movement disorders, fever);

- lab tests (i.e. leukocytes, leukocyte formula, LBP). 

The intra-operatory result the surgeon settled by direct observation during the intervention consisted in assigning each patient to one of the following appendicitis classes: catarrhal, phlegmonous, gangrenous, with possible additional clarifications regarding the occurrence of peritonitis or perforation. The anatomopathological diagnosis was decided based on the histopathological exam which divided the excision specimens into three groups, as follows: catarrhal, phlegmonous and gangrenous. 

Those susceptible of having a second infection source – other than the one caused by appendicitis – were expelled. The rationale for the expel criteria is that we intended to seize the dynamics of change of the biological inflammatory markers since the admission moment up to 72 hrs after surgery, on condition that the unique infection source – caused by appendicitis – be eliminated.

## Results

Out of the 147 patients included in the sample, 79 (53,7%) were females and 68(46.3%) males. 54,4% were catarrhal appendicitis, 32,7% phlegmonous and 12,9% gangrenous (**Table 1**).

**Table 1:**
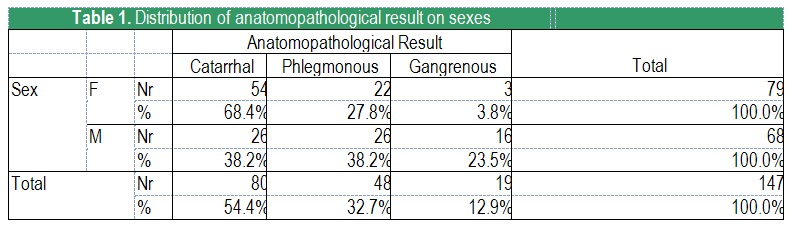
Distribution of anatomopathological result on sexes

**Graph 1:**
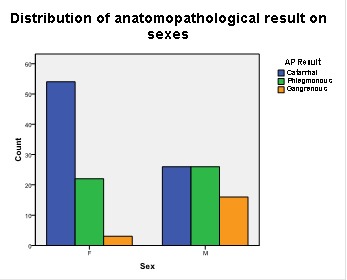
Distribution of anatomopathological result on sexes

The distribution of catarrhal, phlegmonous and gangrenous appendicitis differs significantly statisticwise with SEX (p<0,001; Pearson’s test χ2). It is noteworthy that with female patients 68% of the cases are catarrhal appendicitis, and only 3.8% are gangrenous, while with male patients the percentages are different: there are significantly more cases of gangrenous appendicitis and less catarrhal ones (23.5% and 38.2%, respectively) (**Graph 1**).

The age distribution (see **Table 2**) differs significantly with the AP Result (p<0.001; the ANOVA test). It is easy to notice that the result ”gangrenous appendicitis” usually associates with higher ages. Concretely, the average age of the patients is maximum in the case of gangrenous appendicitis, whereas for the catarrhal and phlegmonous ones it is 14-15 years as low. 

**Table 2:**
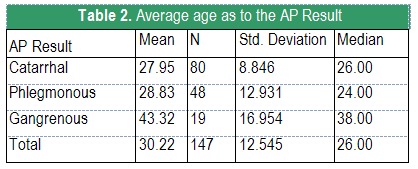
Average age as to the AP Result

**Graph 2:**
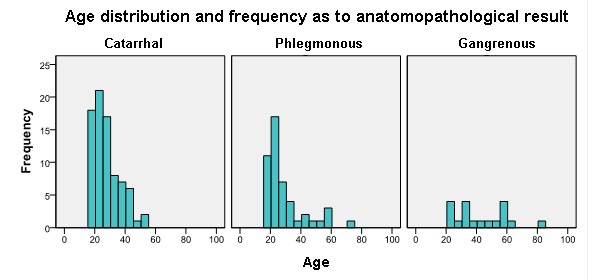
Age distribution (frequency) as to the Anatomopathological Result

Graph 2 leads to the same conclusions, but highlights a different aspect, i.e. the majority of patients with catarrhal and phlegmonous appendicitis are aged between 18-30, whereas the patients with gangrenous ones are relatively evenly distributed between 20-60 years of age. 

**Graph 3:**
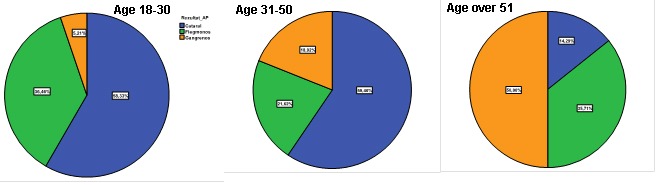
Distribution of the anatomopathological result as to the age groups

Graph 3 shows the distribution of the anatomopathological result as to the age groups 18-30, 31-50 and over 51. 

Table 3. Preoperative LBP, number of Leukocytes, Neutrophiles and Alvarado Score are significantly different, as to the acute appendicitis type inflammation. The increasing order of the average values is the same for all laboratory tests: Catarrhal < Phlegmonous < Gangrenous. 

The only exception from the previous statement refers to Neutrofiles. Neutrofiles’ Frequency is significantly lower in the case of catarrhal appendicitis than in the case of phlegmonous and gangrenous ones, but the values for these last two forms does not differ significantly. 

Leukocytosis with neutrophilia occurs in 100% of acute gangrenous appendicitis (**Table 3**). 

**Table 3:**
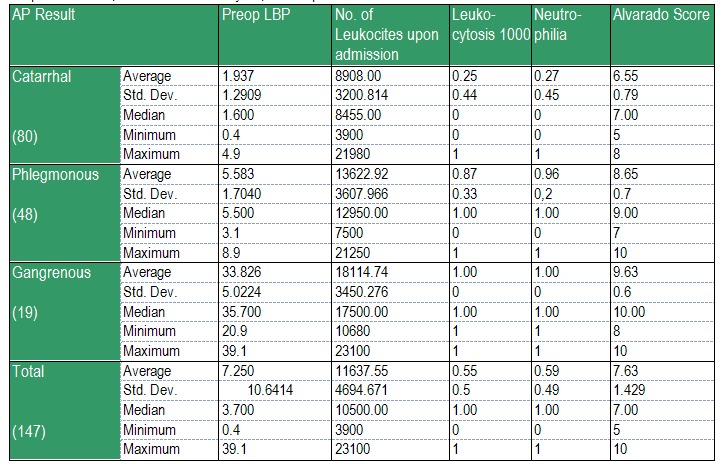
Preoperative LBP, number of Leukocytes, Neutrophiles and Alvarado Score

Table 4 shows the main statistical indicators for the post-op laboratory tests. The following conclusions can be drawn: 

- The mean values of LBP, Leukocyte number are significantly different, with the acute appendicitis type inflammation. The increase order of the mean values is the same for all tests: Catarrhal < Phlegmonous < Gangrenous;

- The only exception to the previous statement refers to LBP: unlike the difference between the means which was very big pre-op, in post-op this difference becomes insignificant between the phlegmonous and the gangrenous form. 

**Table 4:**
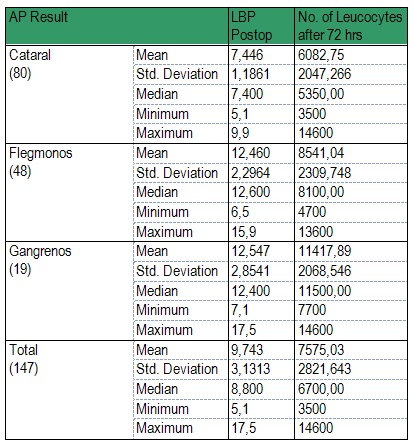
Main statistical indicators for the post-op laboratory

Table 5. Unidimensional statistical analysis of the pre- and post-op evolution of the biological inflamatory markers with patients in the study sample as to the anatomo-pathological type of appendicitis (Catarrhal, Phlegmonous, Gangrenous) (**Table 5**).

 The values of LBP and Leukocytes Number change significantly between pre-op and post-op (p<0,001; t test). This can be seen for all 3 anatomo-pathological types (**Table 5**).

**Table 5:**
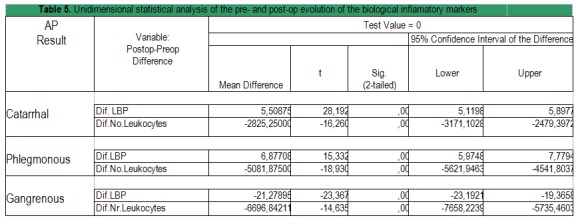
Unidimensional statistical analysis of the pre- and post-op evolution of the biological inflamatory markers

**Graph 4:**
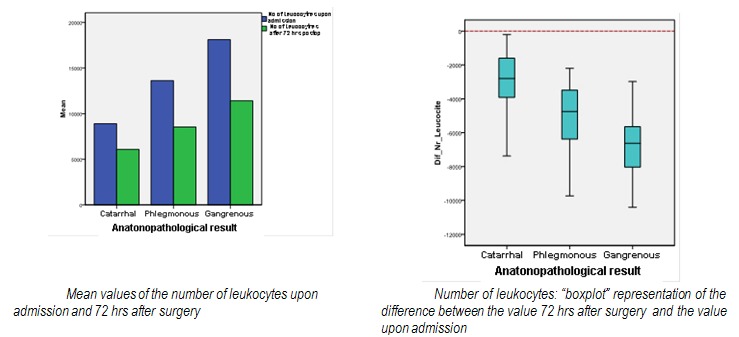
Number of leukocytes evolution between admission value and 72 hrs after surgery

The values of the Leukocytes Number decrease significantly after surgery (**Graph 4**). 

**Graph 5:**
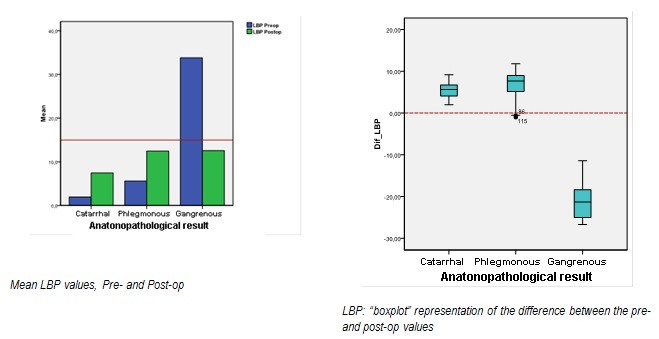
LBP evolution between admission and post-op values, for the three appendicitis types

Also after surgery, the LBP values increase significantly with the catarrhal and phlegmonous types, but decrease significantly with the gangrenous one (**Graph 5**). 

## Discussion

LBP is a biological marker with high pronostic value in the acute appendicitis. LBP dosage pre- and post-op is useful for discrimination among the three types of appendicitis; its recovery to the range statistically considered as normal confirms the infectious process in the organism was solved. 

An interesting remark is that among the biological inflammatory markers dosed in the present study, LBP has a dynamics of its own in catarrhal and phlegmonous appendicitis. Thus, if after the surgical removal of the infection source the leukocytes and neutrophiles values decrease 72 hrs post surgery, LBP continues an ascending curve. A statistically significant decrease of post-op LBP is seen though in the cases of gangrenous appendicitis (in which the pre-op values were significantly higher). 

An explanation to this particular response mechanism is the LBP production mechanism: i.e. by the genic transcription adjustment of the IL1 and IL6-mediated proteic synthesis. The LBP synthesis might be not only connected to the infectious factor’s presence, but also to the level of inflammation in the human body. 

Another possibility might be that the surgical act itself and the post-op wound healing, could stimulate the LBP synthesis by increasing the level of the specific inflammatory mediators, although this stimulation does not reach the extremely increased level seen in the case of gangrenous appendicitis. 

The importance of the present study consists in the introduction of the last generation LBP-type inflammatory markers in the cecal appendix pathology, an extremely frequent occurrence in the emergency surgical pathology, but whose clinical-paraclinical picture is polymorphic, raising doubts in the pre-op diagnosis – especially at the onset of its evolution. This implementation is brand new in the Romanian surgical practise. 

The good correlation between the LBP pre- and post-op values and the hystopathological diagnosis of the appendicitis form we have identified and presented in this study gives the opportunity to use LBP biochemical dosage on a large scale in the management of acute appendicitis. 

Disclosure: No financial interests 
